# An *In Vitro* Study of the Intervertebral Disc Structure Using 3 T Magnetic Resonance Imaging

**DOI:** 10.1097/BRS.0000000000002958

**Published:** 2019-01-15

**Authors:** Vithanage N. Wijayathunga, Steven F. Tanner, John P. Ridgway, Ruth K. Wilcox

**Affiliations:** ∗ Institute of Medical and Biological Engineering, School of Mechanical Engineering, University of Leeds, Leeds, UK; † National Institute for Health Research Leeds Biomedical Research Centre, Chapel Allerton Hospital, Chapeltown Road, Leeds, West Yorkshire, UK; ‡ Department of Medical Physics & Engineering, Leeds Teaching Hospitals Trust, Bexley Wing, St. James University Hospital, Leeds, UK.

**Keywords:** annular fissures, annulus fibrosus, annulus lamellae, gradient echo, granular tissue, high-field magnetic resonance imaging, image quality, intervertebral disc, nucleus pulposus, relative contrast, signal to noise, spin echo

## Abstract

**Study Design.:**

An *in vitro* magnetic resonance imaging (MRI) study.

**Objective.:**

Investigate the potential of high-field MRI for producing higher quality images of the intervertebral disc (IVD) to better distinguish structural details.

**Summary of Background Data.:**

Higher spatial and contrast resolution are important advantages when imaging the complex tissue structures in the spine such as the IVDs. However, at present it is challenging to capture the substructural details in the IVD such as the lamellae.

**Methods.:**

Three MRI sequences; two-dimensional proton-density-weighted Turbo-Spin-Echo (PD-TSE), 2D T2-weighted Turbo-Spin-Echo (T2W-TSE) with fat-saturation (FS), and 3D Spoiled-Gradient-Echo (3D-GE), were modified based on the image quality and scan duration. IVDs of three intact cadaveric lumbar-spines (T12–S1, Age 83–94 yr) were imaged using these optimized sequences. Thereafter each IVD was transversely sectioned and the exposed surfaces were photographed. Landmark observations from corresponding MRI slices and photographs were compared to confirm the MRI captured morphology. The image quality was evaluated using signal-to-noise ratio (SNR), and relative-contrast values. Finally, the underlying tissue structures, including specific pathological features, were qualitatively compared between the MR images and photographs.

**Results.:**

Observations from photographs and corresponding MRI slices matched well. The PD-TSE sequence had better overall SNR, but the relative contrast between the tissue types was relatively poor. The 3D-GE sequence had higher relative contrast between the IVD and bone, but not between annulus and nucleus regions. The T2W images provided the best relative contrast between the annulus and nucleus, however the standard deviations here were high. Structural details including fissures, vascular and granular tissue proliferation, and pathologies in the endplate region, were identifiable from the MR images obtained using the optimized sequences.

**Conclusion.:**

The results demonstrate the potential of high-field MRI to capture the IVD structural details. Since the acquisition durations were within clinically acceptable levels, these methodological improvements have the potential to enhance clinical diagnostics.

**Level of Evidence:** 4

## Abstract 2

Supplemental Digital Content is available in the text

The potential of 3 Tesla magnetic resonance imaging (MRI) to obtain higher quality 2D/3D data-sets of the intervertebral disc (IVD) has been investigated using cadaveric IVDs and three clinical imaging protocols optimized based on image quality. Details of the IVD substructure including annulus and endplate features were seen better than what is normally seen in routine clinical MRI.

Technological advances in magnetic resonance imaging (MRI) are having a significant impact on clinical diagnostics. Even though the capital costs are larger, clinical imaging at higher field strengths is becoming increasingly common due to the improved signal-to-noise ratio (SNR) alongside the availability of new coil designs and more versatile imaging sequences. The SNR of a MRI imaging system operating at a field strength of 3 Tesla (3 T) is approximately twice that of a 1.5 T system at similar imaging conditions. This relative increase in SNR can be utilized to reduce the scan duration or to improve the image resolution without compromising image quality. Reduced scan durations can increase the patient throughput, while helping to reduce motion artefacts in longer imaging sequences.

Higher spatial resolution and improved contrast are important advantages for imaging complex tissue structures in the spine such as the intervertebral disc (IVD). The IVD comprises multiple, structurally distinctive constituents that have the potential to influence image contrast. In healthy spines, the hydrated nucleus pulposus (NP) primarily consists of water (70–90% of wet weight), proteoglycans (65% of dry weight), randomly oriented collagen (15–20% dry weight),^1^ and elastin (∼2% dry weight^2^). The annulus fibrosus (AF) contains multiple, orderly structured lamellae of collagen (65–70% of dry weight), and the fiber orientation exhibit a criss-cross pattern in these alternating layers. Water (∼70% wet weight), proteoglycans (10–20% of dry weight),^3^ and elastin are other key constituents in the AF. In the IVD endplate (EP), cartilaginous and bony components will influence the image contrast.

Although different imaging sequences are possible for spinal MRI, the selection is primarily driven by the clinically relevant structural and pathophysiological details that need to be investigated. However, at present it is very difficult to capture the structural details within the IVD such as the AF lamellae, when using routine clinical imaging conditions. Further, with the increased strength in the static magnetic field at 3 T, there come added challenges such as greater local magnetic field in-homogeneities caused by higher magnetic susceptibility differences, larger chemical shift effects, and limitations imposed by higher specific absorption rates (SAR). The SAR value estimates the radiofrequency energy absorbed by the tissue and hence the potential for tissue heating resulting from a clinical MRI examination.

Therefore the aim of this study was to investigate the potential of high-field 3 T MRI to obtain higher quality 2D and 3D data sets of the IVD than 1.5 T MRI, specifically for better visualization (both resolution and contrast) of clinically-relevant details such as endplate defects, annulus fissures and delamination, inflammatory and Modic changes, and revascularization. Three potentially suitable protocols were investigated on cadaveric spines, and comparisons were made with high-resolution photographic imaging of the same IVDs.

## MATERIALS AND METHODS

### Sample Preparation

Three cadaveric spines were sourced from the Leeds GIFT Research Tissue Project after ethical approval (two male, one female, age 83–94 yr, degeneration levels 4–8 as per Modified Pfirrmann Grading^4^). From each spine, the lumbar segment was separated by dissecting across the T12 and S1 vertebrae. Excessive soft tissue around the column was removed leaving sufficient to preserve the IVDs and the vertebrae.

The segments were wrapped in paper towels lightly-soaked with phosphate buffered saline (PBS) in order to avoid dehydration during handling between harvesting and sealing in polymer bags for freezing. PBS was used due to its isotonicity. Subsequently the segments were stored at −20 °C. Prior to MRI scanning, each sample was defrosted overnight at 3 °C and sealed in two layers of MRI-safe transparent polymer lining.

### MR Imaging

MR imaging was performed on a Siemens Magnetom Verio 3 T scanner with a maximum gradient strength of 45 mT/m and a maximum slew rate of 200 T/m/s, at a temperature of 22 ± 0.5 °C. Three different imaging sequences were used: 2D proton-density (PD) weighted Turbo-Spin-Echo (TSE), 2D T2-weighted TSE with fat-saturation (FS), and 3D spoiled-gradient-echo (GE) (Fast-Low-Angle-Shot).

The 2D-TSE and 3D-GE pulse sequences were selected to provide complementary information. The TSE sequences utilize multiple 180° RF refocusing pulses that minimize the effect of magnetic field homogeneities induced by the differing magnetic susceptibilities of the different tissue components, therefore resulting an overall higher signal-to-noise ratio. The absence of the 180° refocusing pulse in the GE sequence results in signal intensities that are influenced by variations in magnetic susceptibility. The absence of the 180° RF pulse and the use of a low flip angle excitation pulse allows a much shorter repetition time to be used for the GE sequence in comparison to the SE sequences, enabling a much higher acquisition matrix to be acquired within a comparable time.

The imaging conditions were refined by considering image “quality” and scan duration in an initial analysis involving both ovine and bovine spine tissue (results presented in Supplementary Data; http://links.lww.com/BRS/B406). The best imaging parameters identified by this process (shown in Table 1) were then applied to the cadaveric specimens examined in this study.

**TABLE 1:** Values Selected for the Key Imaging Parameters of the Three Sequences

Scan Parameter	2D TSE PD-Weighted Sagittal	2D TSE T2-Weighted Fat Saturated Sagittal	3D GE (FLASH) Sagittal
Repetition time TR, ms	3520	3520	11
Echo time TE, ms	12	63	3.7
Flip angle	153	147	20
Averages	1	1	1
Pixel bandwidth	250	199	250
Echo train length	5	5	1
Width × height, mm	160.0 × 160.0	160.0 × 160.0	160.0 × 160.0
Acquisition matrix (rows × columns)	512 × 512	512 × 512	512 × 512
Slice thickness, mm	1.2	1.2	1.2
Number of slices	36	36	36
Space between slices, mm	1.44	1.44	–
Acquisition time, min	6.01	6.0	3.23
Resolution (pixels per mm)	3.2	3.2	3.2
Voxel size, mm	0.3125 × 0.3125 × 1.2	0.3125 × 0.3125 × 1.2	0.3125 × 0.3125 × 1.2
Chemical shift, pixels	∼1.8	∼2.5	∼1.8

The chemical shift was calculated using the pixel bandwidth (pxBW) and resonance frequency difference between fat and water at 3 T (∼447 Hz). TSE indicates Turbo-Spin-Echo.

The cadaveric spine segments were placed on the scanner bed, at the magnet iso-center, in the head-first-supine orientation. Sagittal and transverse MR-images of the IVD were obtained using the imaging conditions summarized in Table 1. The image distortion correction algorithm provided by the scanner manufacturer was incorporated within these acquisition settings. The SAR value was also recorded for each scan. Extensively collapsed discs were not scanned, and in total 11 IVDs from the three spines were imaged. Since each sample had undergone a number of freeze-thaw cycles after harvest, there was some redistribution in the fluid/fat within the vertebral body. This redistribution was reflected in the MRI data and resulted in a linear pattern of hyper-intense signals within the vertebral bodies. Therefore randomly selected samples were imaged using micro-computed tomography to confirm the trabecular integrity.

Thereafter, using the image datasets obtained for different sequences, quantitative and qualitative observations were made as described below.

### Image Quality Measurement

In order to compare the image quality from the MR sequences, the SNR was measured using image processing software (ImageJ, NIH, Maryland). Separate regions of interest (ROI) were selected within the anterior and posterior annulus, nucleus, and vertebral bone. Elliptical areas were selected on one slice (allowing as much coverage as possible) and copied through five consecutive slices in the image stack, checking to ensure that the selected areas remained within the tissue under consideration. Due to the presence of large fissures in some of the regions, which differed in signal intensity from the surrounding tissue, the analysis was repeated with these regions excluded. There was insufficient thickness to obtain meaningful values from the vertebral endplates, so these regions were not evaluated.

The mean value of the signal intensity for each of these ROI was calculated. The largest possible rectangular region outside the IVD in the image background (air) was also selected, and the standard deviation of the background noise intensity was calculated. The SNR for each of the ROIs was then determined by dividing its mean signal intensity (*S*) by the standard deviation of the background noise intensity.^5^

The Relative Contrast (ReC) between tissue regions was calculated as 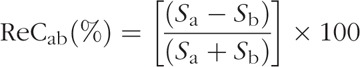


where the subscripts a and b denote the two regions.

### Image Artefact Assessment

The chemical shift artefact represents a spatial mis-registration of fat tissue relative to water tissue in the frequency-encoding direction. In order to assess the susceptibility to chemical shift artefacts, the sagittal sequences were repeated with phase encoding direction changed from Anterior-Posterior to Head-Feet, and the transverse sequences repeated with phase encoding direction changed from Anterior-Posterior to Right-Left.

The assessment of chemical shift and aliasing (“wrap”) artefacts were carried out for all sequences and in data acquired from all of the specimens.

### Comparison of MR Images and Section Photographs

Following imaging, each sample was held in a custom made rig and then transversely sectioned through the IVD with a single incision using a post-mortem knife. Both exposed surfaces of the IVD were photographed using a high-resolution digital camera (Canon EOS 550D, EF 100 mm f2.8 macro lens) so that structural features, such as the major lamellae and any fissures, were captured.

The photographs of each sectioned IVD plane and the corresponding MR images (transverse) were imported to ImageJ software, and three clear landmarks that appeared on both the MRI and photograph were identified. The distances between the landmarks were measured and the difference between photograph and MRI were determined.

Finally, the underlying tissue structures, including specific pathological features, were qualitatively compared between the MR images and photographs.

## RESULTS

### Image Quality Measurement

The image quality metrics determined from the image stacks obtained under each optimized sequence are presented in Tables 2 and 3. It was found that the PD-TSE had better overall SNR than the other sequences (Table 2) however, this sequence gave the poorest relative contrast between the tissue types (Table 3). The 3D-GE sequence had higher relative contrast between the IVD and bone, but not between AF and NP regions. The T2W-TSE images provided the best relative contrast between the NP and AF, however the standard deviations here were high.

**TABLE 2:** Signal-to-Noise Ratio (SNR) Calculated from the MRI Images of the Three Sequences, for the Different Regions in the IVD

Sequence	Tissues	Mean SNR (±SD) Considering all the Imaged Samples	Mean SNR (±SD) Excluding Samples with Major Fissures
2D PD TSE sagittal	Nucleus	35.5 (±3.7)	31.1 (±3.8)
	Anterior annulus	37.8 (±22.9)	25.7 (±13.1)
	Posterior annulus	27.3 (±4.5)	29.7 (±2.3)
	Vertebral bone region	39.2 (±4.9)	36.4 (±0.9)
2D T2-weighted TSE fat saturated sagittal	Nucleus	14.6 (±4.5)	12.1 (±1.5)
	Anterior annulus	11.3 (±9.1)	6.1 (±2.5)
	Posterior annulus	6.3 (±3.7)	8.0 (±3.2)
	Vertebral bone region	12.1 (±0.5)	12.1 (±0.6)
3D GE (FLASH) sagittal	Nucleus	20.8 (±4.5)	18.6 (±3.6)
	Anterior annulus	35.4 (±16.8)	20.8 (±9.8)
	Posterior annulus	17.6 (±1.4)	17.6 (±2.0)
	Vertebral bone region	8.4 (±0.7)	8.6 (±0.9)

Standard deviation (SD) of the calculated values is indicated within the brackets.

**TABLE 3:** Relative Contrast (ReC) Percentages Calculated from the MRI Images of the Three Sequences, for the Different Regions in the IVD

Sequence	Tissues Contrasted	Mean ReC (±SD) Considering all the Imaged Samples	Mean ReC (±SD) Excluding Samples with Major Fissures
2D PD TSE sagittal	Nucleus—anterior annulus	3.4 (±27.3)	16.8 (±20.2)
	Nucleus—posterior annulus	13.1 (±11.2)	6.7 (±1.7)
	Nucleus—vertebral bone region	−4.9 (±3.9)	−3.5 (±4.4)
	Anterior annulus—vertebral bone region	−7.9 (±26.9)	−20.0 (±24.0)
	Posterior annulus—vertebral bone region	−17.8 (±13.3)	−10.2 (±2.6
2D T2-weighted TSE fat saturated sagittal	Nucleus—anterior annulus	21.0 (±24.2)	34.0 (±12.5)
	Nucleus—posterior annulus	39.5 (±31.3)	22.4 (±13.5)
	Nucleus—vertebral bone region	7.7 (±14.4)	0.1 (±8.8)
	Anterior annulus—vertebral bone region	−13.1 (±38.0)	−33.4 (±20.2)
	Posterior annulus—vertebral bone region	−35.1 (±27.5)	−22.0 (±21.7)
3D GE (FLASH) sagittal	Nucleus—anterior annulus	−22.6 (±13.6)	−16.3 (±11.4)
	Nucleus—posterior annulus	7.4 (±13.7)	2.6 (±15.1)
	Nucleus—vertebral bone region	41.8 (±9.2)	36.7 (±3.9)
	Anterior annulus—vertebral bone region	57.6 (±16.3)	49.6 (±12.2)
	Posterior annulus—vertebral bone region	35.6 (±6.9)	34.4 (±9.3)

Standard deviation (SD) of the calculated values is indicated within the brackets.

### Image Artefact Assessment

There were no aliasing artefacts in any of the images. When the phase encoding direction was swapped, there was very little distortion or local change in signal appearance due to chemical shift effects.

### Comparison of MR Images and Section Photographs

**Figure 1:**
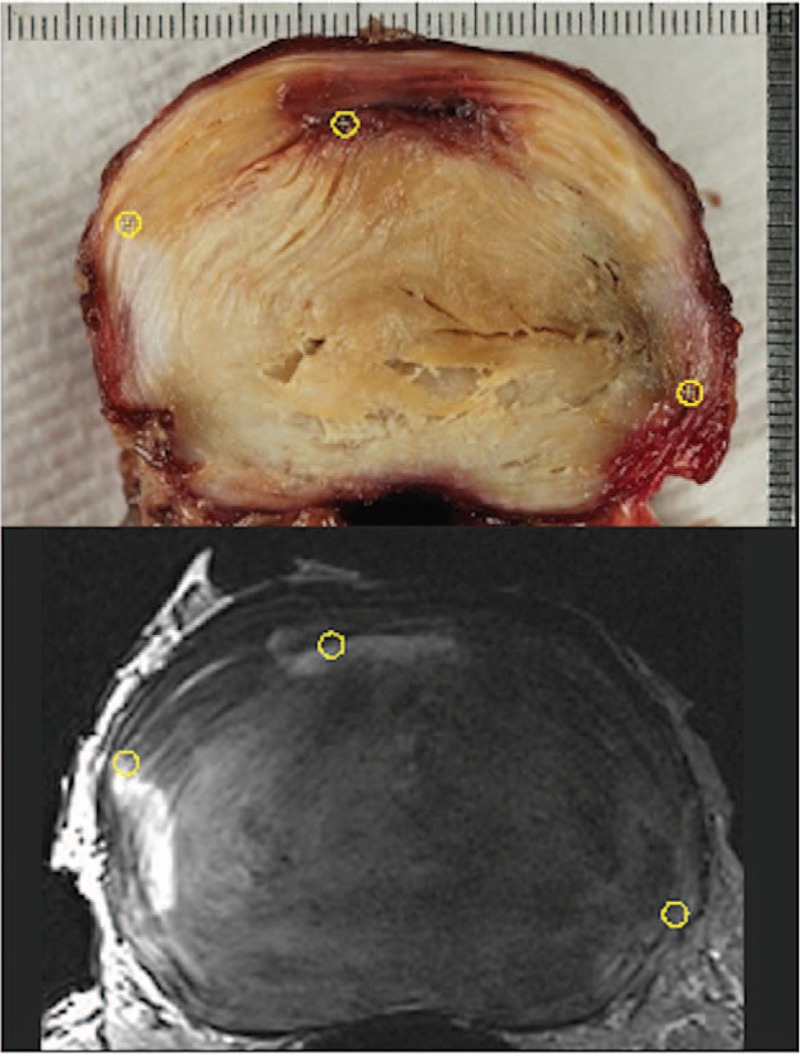
Example comparison of landmarks from an MR image (in this case T2-weighted-TSE) and corresponding cross-sectional photograph of a L2L3 IVD. On the MR image, the fluid build-up, vascularization, and the granulation tissue proliferation due to fissures are indicated well and clearly distinguishable from the surrounding tissue. IVD indicates intervertebral disc; TSE, Turbo-Spin-Echo.

A comparison of the transverse MR images and the cross-sectional photographs of equivalent IVDs showed many common features in both image modalities (Figure 1). Further, relatively small differences were found between inter-landmark length measurements from MR and corresponding photographs (Table 4), indicating good compatibility of landmarks locations.

**TABLE 4:** Range of Length Measurement Difference Between Section Photographs and MR Images Expressed as a Percentage in Relation to the Length Measured from the Photograph

Lowest Length Difference (%)	Highest Length Difference (%)	Median Length Difference (%)	Root Mean Square Length Difference (%)
−8.8	5.2	−0.5	3.634

### IVD Structural Features from the MR Images

**Figure 2:**
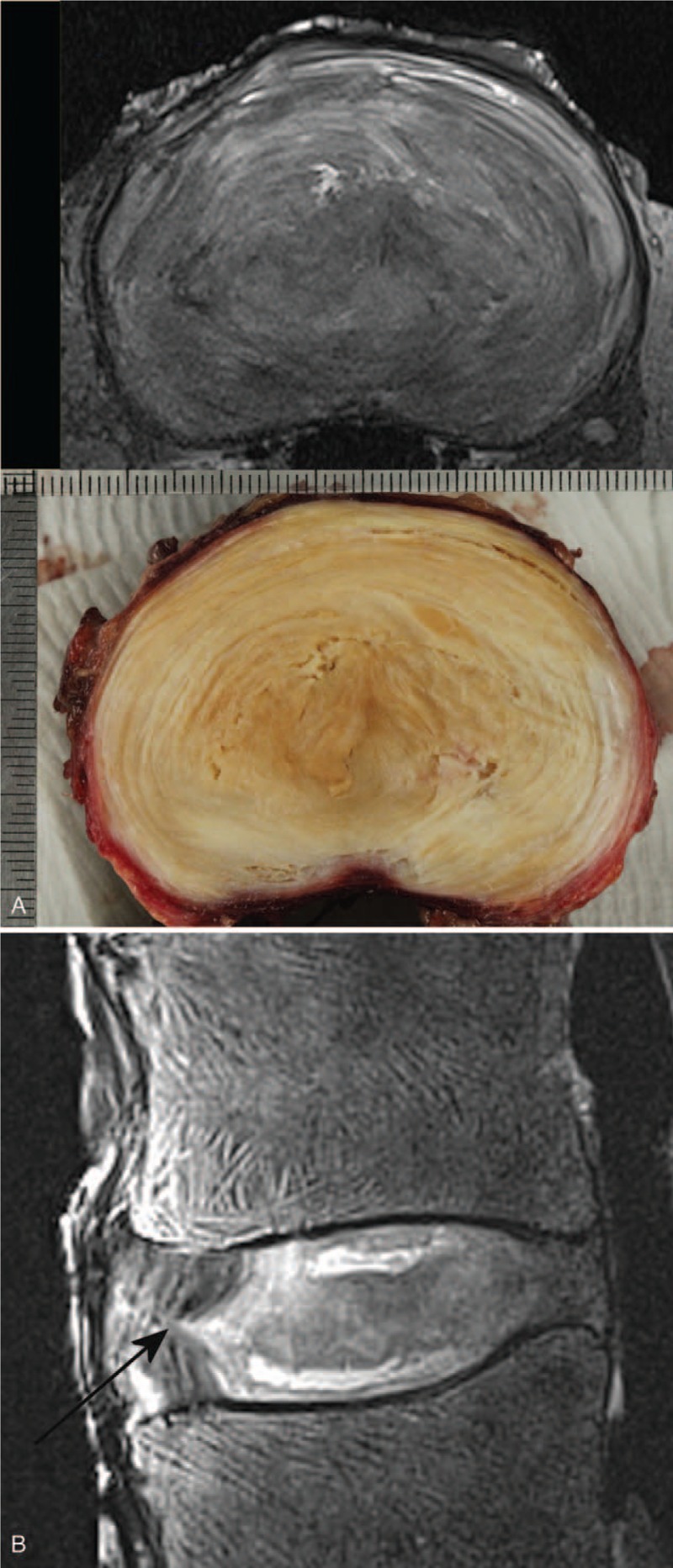
(**A**) Photograph of dissected section compared with 2D PD TSE transverse MR slice, and (**B**) 2D T2-weighted TSE fat saturated sagittal MR image of a L1L2 IVD. The anterior lamellae are drawn into the annular fissure (indicated by the arrow) in the sagittal plane (right anterio-lateral in the transverse plane). The fibrotic nucleus appears less intense, and fissures in the nucleus closer to the endplates appear bright. The vertebral-fluid rearrangement due to repeated freeze-thaw cycles are seen as a linear pattern of hyper-intense signals within the vertebral bodies on the sagittal image. IVD indicates intervertebral disc; TSE, Turbo-Spin-Echo.

**Figure 3:**
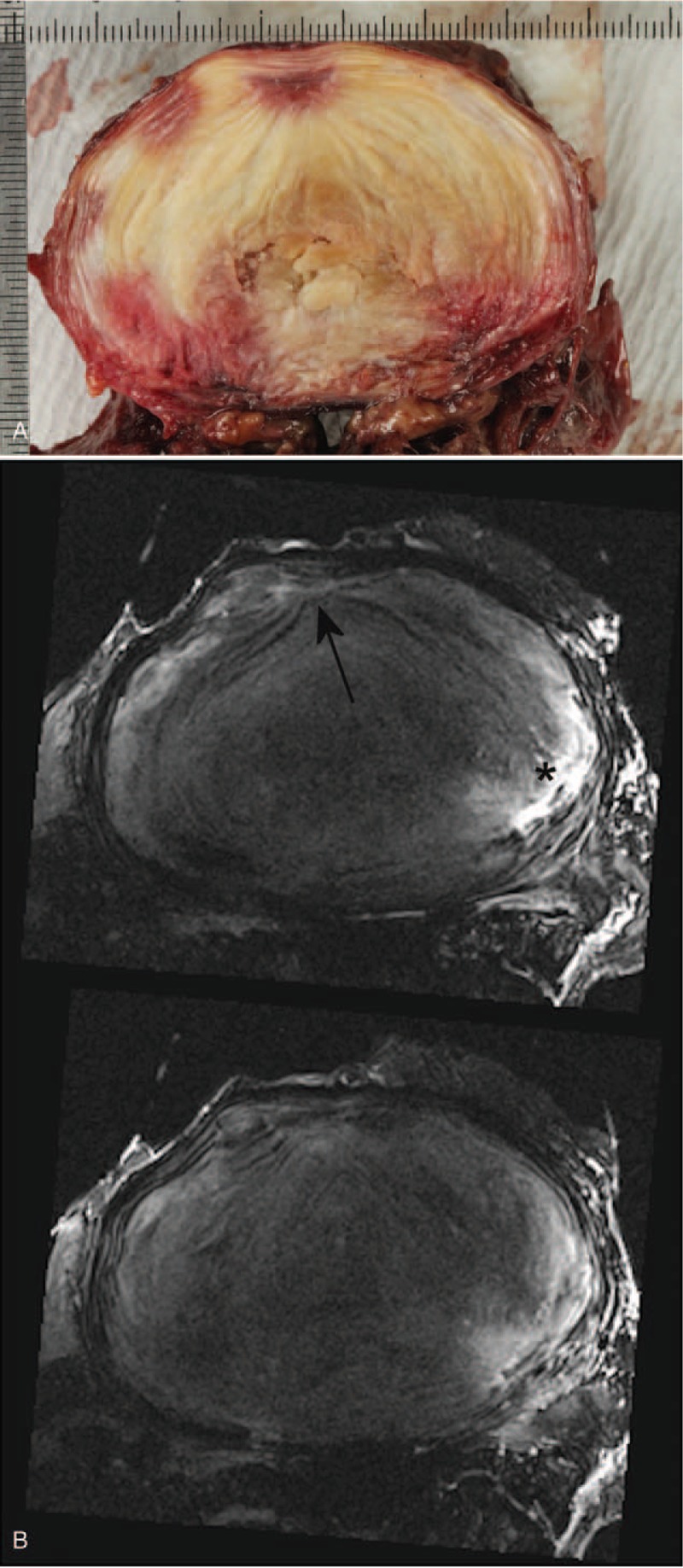
Transverse section photograph (**A**) and 2D T2-Weighted TSE fat saturated transverse MR-images (**B**) of a L4L5 disc showing the lamellae structure getting drawn in to the fissure (indicated by the arrow). Vascularization and fluid build-up (indicated by the ∗) are also clearly seen. The nucleus is almost entirely fibrotic giving a much lower signal. TSE indicates Turbo-Spin-Echo.

**Figure 4:**
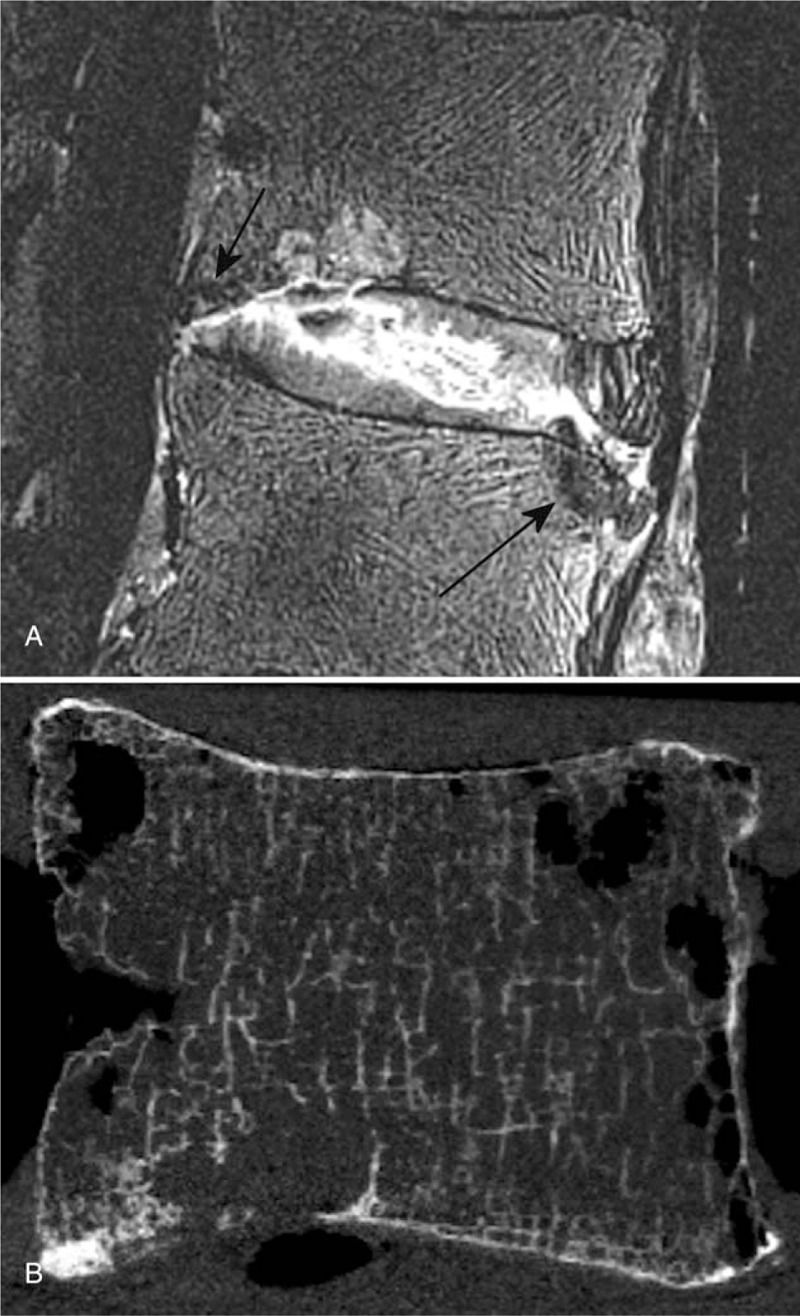
2D T2-weighted TSE fat saturated sagittal MR Image (**A**) of a L2L3 disc showing a fissure completely across the disc with endplate disruption, vascular proliferation deep in to the nucleus, Schmorl node, and hypo-intense (sclerotic) developments in; caudal endplate anterior region and cranial endplate posterior region (indicated by arrows). Anterior lamellae that are intact are clearly visible on the image. The vertebral-fluid rearrangement due to repeated freeze-thaw cycles are seen as a linear pattern of hyper-intense signals on the sagittal image. A sagittal slice from a micro-Computed Tomography (μCT) scan performed on the same L2 vertebra after dissecting it out following MR imaging to assess the trabecular integrity, is shown on **B**. The μCT slice is approximately from the same sagittal region as of the MR image. TSE indicates Turbo-Spin-Echo.

Structural details within the tissue constituents were identifiable from the MR images. In particular, the lamellae structures were visible and seen to be disturbed and drawn towards annular fissures (Figures 2A, B and 3A, B). Granular tissue and vascular formation due to fissures were seen hyper-intense in both T2W and PD sequences (Figures 1 and 2). The fibrotic regions, due to loss of proteoglycans, appeared hypo-intense (Figure 3A, B). Further, the extent of the Schmorl nodes and sclerotic formations in the endplate-region were well defined (Figure 4A, B).

## DISCUSSION

The IVD consists of multiple tissue constituents organized in a complex structural arrangement. The natural process of aging as well as various pathological processes will have significant effect on the IVD structure, resulting in many changes to its arrangement, morphology, and composition. MRI is probably the favored modality to image the structural and morphological presentation of the soft tissues, and it has been the basis of several classification systems for disc degeneration.^4,6,7^ The majority of these grading systems focus on T2-weighted sagittal scans.

Here, three sequences that have previously found use when imaging the spine at 1.5 T have been modified for use at 3 T. These modifications were directed at implementing a set of imaging conditions that could be translated to *in vivo* applications at the higher field strength to acquire data with adequate image contrast, SNR, and resolution. Image fidelity was assessed in the 3 T MR data by comparison with equivalent photographic images acquired from the same specimens. The MR imaging parameters were chosen on the basis of a number of compromises that included improving the image resolution at the “expense” of SNR. However, if there is sufficient relative contrast between the structures of interest, the lack of SNR could be somewhat mitigated. Under such circumstances the images may look grainy (noisy), yet might provide better insight to the underlying structural characteristics.

**Figure 5:**
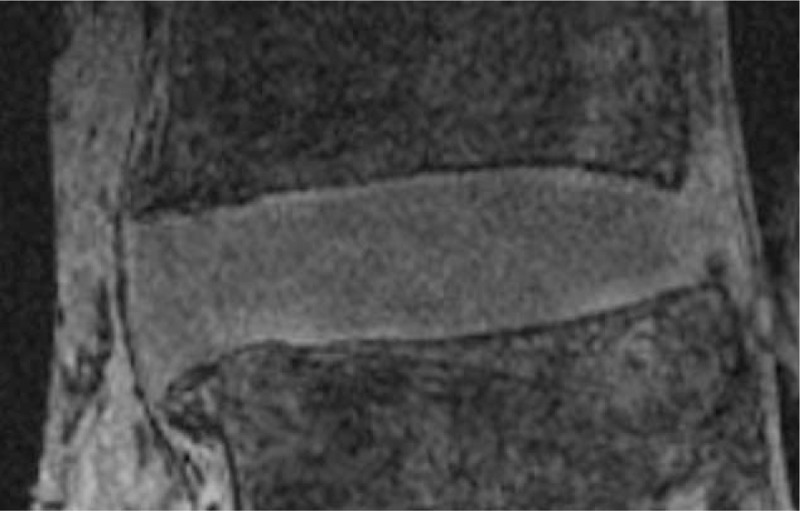
3D GE (FLASH) sagittal image of a L3L4 disc. The relative contrast of the IVD with respect to the vertebral bone is higher. IVD indicates intervertebral disc.

The results of this study reflected these differing image characteristics. From the quantitative results presented in Tables 2 and 3, the PD TSE sequence was found to have the best overall SNR, making it a good option for capturing the gross anatomy, however the contrast between tissue types was poorer. The T2W TSE had lower SNR due to the long TE; this sequence provided the best relative contrast between the nucleus and annulus, but the larger standard deviation values indicate that the sequence is very sensitive to the condition (structural and biochemical) of the disc. There was low relative contrast between the nucleus and bone region, but the dark appearance of the bony endplate enables the two tissues to be visually separated. The GE sequence had higher relative contrast between IVD and bone region, but not between the annulus and nucleus (Figure 5). Further, the effects of susceptibility artefacts were negligible, and no other artefacts such as aliasing were observed. Protons in fat and water resonate at slightly different frequencies due to the differences in their electron environments. As a result, fat containing structures are shifted along the frequency encoding direction relative to the signals emanating from the tissue water. At 3.0 T the difference in resonance frequencies is about 447 Hz and based on the imaging parameters used, the relative shift occurring in the images acquired in this study was between approximately 0.56 and 0.79 mm. Chemical shift in the IVD mainly arises at the endplate-vertebral bone-marrow margin. However the use of the fat-saturation pulse with T2W TSE sequence allowed to reduce the higher chemical shift associated with that sequence. Further, the frequency-encoding was selected not to coincide with head-feet direction, in order to minimize the chemical shift on the sagittal images.

The annular fissures, which are disruption or separation of fibers, fiber bundles or lamellae in the AF, cause inflammation, leading to granulation tissue, vascularization (particularly when the fissure is in the outer AF), and increased presence of fluid. If the disruption is connected to the NP it allows mucinous material from the NP to flow in.^8–10^ These processes will give rise to a high intensity zone (HIZ) on T2-weighted MR images.^11^ The contrast of the HIZ will depend on the fissure size, extent, and stage of its progression, as well as on the imaging characteristics such as resolution, SNR, CNR, and ReC.^12^ Therefore only a minority of fissures are effectively picked up on MRI.

Well-developed fissures in the IVD significantly affect the image-quality measures, as was seen here particularly in the anterior annulus, as evidenced by the higher standard deviations in signal in this region. When samples with heavy fissuring were removed from the image quality analysis, the standard deviations for the annulus reduced considerably.

The main difficulties associated with the comparison of sectioned plane photographs and the corresponding transverse MR images arise due to the IVD dissection plane and the MRI imaging plane not being exactly the same, and there may be tissue disturbance due to the knife traverse, particularly in the heavily degenerated discs. However, as shown in Table 4, the root mean square error for the length measurements compared between photographic and MRI data sets, based on structural landmarks, is less than 4%. This confirms that the structural features identified on MR images match well with the actual.

Most spinal segments imaged in this study exhibited considerable degenerative features, providing the opportunity to test the sequences in a more clinical sense. As seen from Figure 2, when the annular architecture was reasonably intact, the sequences in this study were able to pick up the lamellae patterns. This is useful, as it helps to identify the annular fissures, and their influence on the structural arrangement. The lamellae appear to be drawn into the fissure, creating a cone-shaped arrangement of fibers in 3D (Figures 2 and 3). This is probably due to the weakened annular structure, and the pressure gradient between the nucleus and the fissure, causing the fluid or mucoid material from the nucleus to be pushed towards the fissure. Chronic inflammation will cause tissue fibrosis resulting matrix remodeling. In the IVD, particularly in the inner regions, this is indicated by an increase in collagen and fibronectin, and a loss of proteoglycans.^13^ Typically, this will give rise to a hypo-intense signal on T2-W and PD MR images for the affected regions in comparison to a healthy disc, as seen in Figures 2 and 3.

MRI changes in the endplate regions were also picked up in detail by the sequences used in this study. In Figure 4, the morphology and the extent of both the Schmorl node and the sclerotic development are visible, with the edge of the affected region distinguishable against the surrounding tissue. Since almost all the samples were degenerated, the GE sequence was unable to pick up a hyper-intense band for the cartilaginous endplate. Yet the bony endplate is visible, allowing the identification of endplate disruptions.

Even under high-field MRI, the challenges in capturing structural variations in the IVDs are substantial. The presence of annular fissures is mainly identified from the observation of hyper-intense zones in either T2W images or contrast-enhanced T1W images.^14,15^ Berger-Roscher *et al*^16^ reported that clinical spin-echo sequences at 3 T were unable to detect fresh lesions created using needle punctures on bovine tail discs from calves and juvenile cattle even though the outcomes at 11.7 T were promising. It highlighted the difficulties in identifying lesions on 3 T even when longer acquisition times were employed. Wijayathunga *et al*^17^ has previously been successful in capturing intricate structural details of the IVD, using both spin echo and gradient echo sequences on a 9.4 T laboratory MR scanner. The research efforts carried out towards translation of those concepts to more clinically amenable imaging conditions (low acquisition times in particular) on a 3 T MR platform formed the basis for this manuscript. In this study, optimal selection of imaging parameters with due consideration to image quality metrics has resulted promising imaging outcomes, albeit under *in vitro* conditions. The chosen imaging parameters have allowed the capture the major lamellae architecture, signal intensity variations for granulation, fibrotic, vascularized, or inflamed tissue with good demarcation of the regions. Importantly the acquisition durations were within clinically acceptable levels. Since MRI technology is a fast improving area, and the feasibility of ultra-high-field MRI is increasing, these methodological improvements have the potential to significantly enhance future clinical diagnostics.

**Key Points**

- It is challenging to capture the substructural details in the IVD such as the lamellae, using routine clinical imaging conditions.
- In this study, three MR imaging sequences (2D PD-TSE, 2D T2W-TSE, and 3D GE-FLASH) were optimized based on image quality and scan duration.
- When cadaveric lumbar IVDs were imaged using the optimized sequences, greater details of the IVD substructure including fissures, vascular and granular tissue proliferation were identifiable from the images, than what is normally seen in routine clinical MRI.
- Since the acquisition durations were within clinically acceptable levels, these methodological improvements have the potential to enhance clinical diagnostics.
